# The Curies’ element: state of the art and perspectives on the use of radium in nuclear medicine

**DOI:** 10.1186/s41181-023-00220-4

**Published:** 2023-11-10

**Authors:** Sara Franchi, Mattia Asti, Valerio Di Marco, Marianna Tosato

**Affiliations:** 1 Department of Chemical Sciences, University of Padova, Via Marzolo 1, 35131 Padua, Italy; 2 Radiopharmaceutical Chemistry Section, Nuclear Medicine Unit, AUSL di Reggio Emilia: Azienda Unità Sanitaria Locale - IRCCS Tecnologie Avanzate e Modelli Assistenziali in Oncologia di Reggio Emilia, Via Amendola 2, 42122 Reggio Emilia, Italy

**Keywords:** Radium-223, Radium-224, Targeted alpha therapy, α-emitters, Radium chelators

## Abstract

**Background:**

The alpha-emitter radium-223 (^223^Ra) is presently used in nuclear medicine for the palliative treatment of bone metastases from castration-resistant prostate cancer. This application arises from its advantageous decay properties and its intrinsic ability to accumulate in regions of high bone turnover when injected as a simple chloride salt. The commercial availability of [^223^Ra]RaCl_2_ as a registered drug (Xofigo^®^) is a further additional asset.

**Main body:**

The prospect of extending the utility of ^223^Ra to targeted α-therapy of non-osseous cancers has garnered significant interest. Different methods, such as the use of bifunctional chelators and nanoparticles, have been explored to incorporate ^223^Ra in proper carriers designed to precisely target tumor sites. Nevertheless, the search for a suitable scaffold remains an ongoing challenge, impeding the diffusion of ^223^Ra-based radiopharmaceuticals.

**Conclusion:**

This review offers a comprehensive overview of the current role of radium radioisotopes in nuclear medicine, with a specific focus on ^223^Ra. It also critically examines the endeavors conducted so far to develop constructs capable of incorporating ^223^Ra into cancer-targeting drugs. Particular emphasis is given to the chemical aspects aimed at providing molecular scaffolds for the bifunctional chelator approach.

## Background

Targeted Alpha Therapy (TAT) is an evolving strategy for cancer therapy that is regarded as an alternative or complementary approach to traditional treatment options such as surgery, chemotherapy, and external beam radiation (Ferrier and Radchenko 2019; Miederer et al. 2008). TAT lies in the selective delivery of a suitable α-particle emitting radionuclide to primary tumors or metastases in order to destroy the malignant cells while sparing the surrounding healthy sites (Makvandi et al. 2018; Radchenko et al. 2021).

**Fig. 1:**
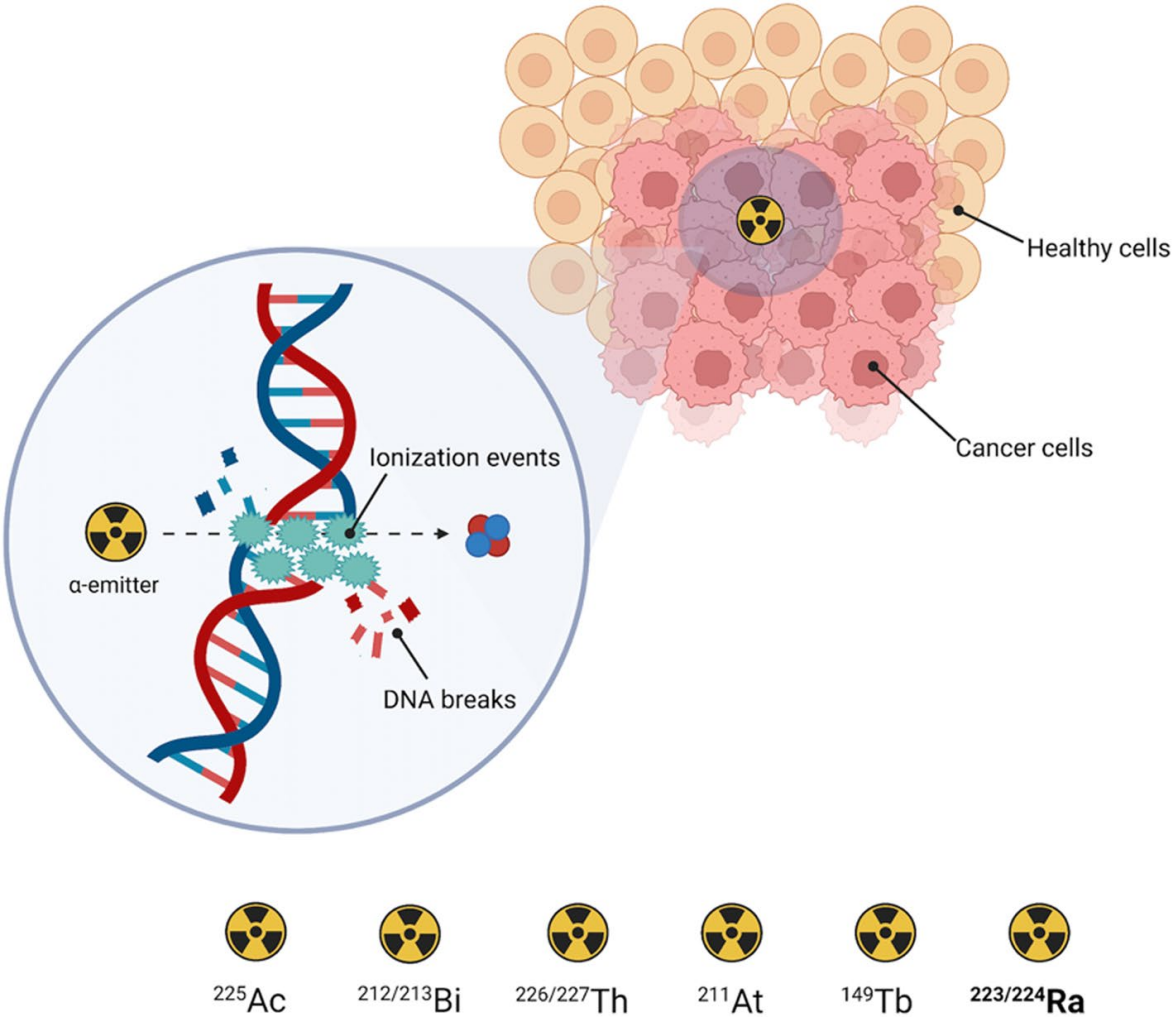
Schematic representation of the effect of α-radiation at cellular and sub-cellular level and list of medically relevant α-emitters. Image created with https://www.biorender.com

α-Particles consist of 2 protons and 2 neutrons (*i.e.* helium-4 nuclei – ^4^He^2+^) and possess a high ionization potential (Curie 1911; Sgouros 2008). Their high linear energy transfer (LET, 80–100 keV/μm) causes the deposition of a massive energy (2–10 MeV *per* particle) over a short path length (50–100 μm, corresponding to a few cellular diameters) inducing irreversible cell damage (Eychenne et al. 2021; Ferrier and Radchenko 2019). In particular, α-particles are able to efficiently trigger non-repairable DNA wreckage through double-strain breaks, thus inducing cell death (Fig. 1) (Eychenne et al. 2021; Ramogida and Orvig 2013).

As a consequence of their physical characteristics, the use of α-particles properly directed to cancer tissues leads to a reduced irradiation of surrounding healthy cells, making this approach particularly favorable and efficient for treating small tumors, disseminated metastases, or isolated cancer cells (Eychenne et al. 2021). Moreover, being the cytotoxicity of α-particles independent of oxygen concentration and dose rate, they provide advantages in the treatment of malignancies featuring hypoxic regions (Franchi et al. 2022; Miederer et al. 2008; Poty et al. 2018; Yang et al. 2022).

Unfortunately, the selective accumulation of a radionuclide in its “free” ionic form (*e.g.*, as a salt) in specific target tissues, organs, or compartments is feasible only in a few cases (Lewis et al. 2019). This does not occur for the vast majority of radiometals, that are the most employed radionuclides for this kind of treatments. As a consequence, a tumor-seeking vector (*e.g.*, small molecule, peptide or antibody) is more commonly used to target cancer biomarkers with high affinity and selectivity and a bifunctional chelator (BFC), covalently attached to the biological vector, is then employed to trap and retain the radiometal in vivo (Boros and Packard 2019; Lewis et al. 2019; Price and Orvig 2014; Ramogida and Orvig 2013; Reubi et al. 2005). This approach allows treating a huge variety of tumor types by tuning the nature and properties of the tumor-seeking molecule.

**Fig. 2:**
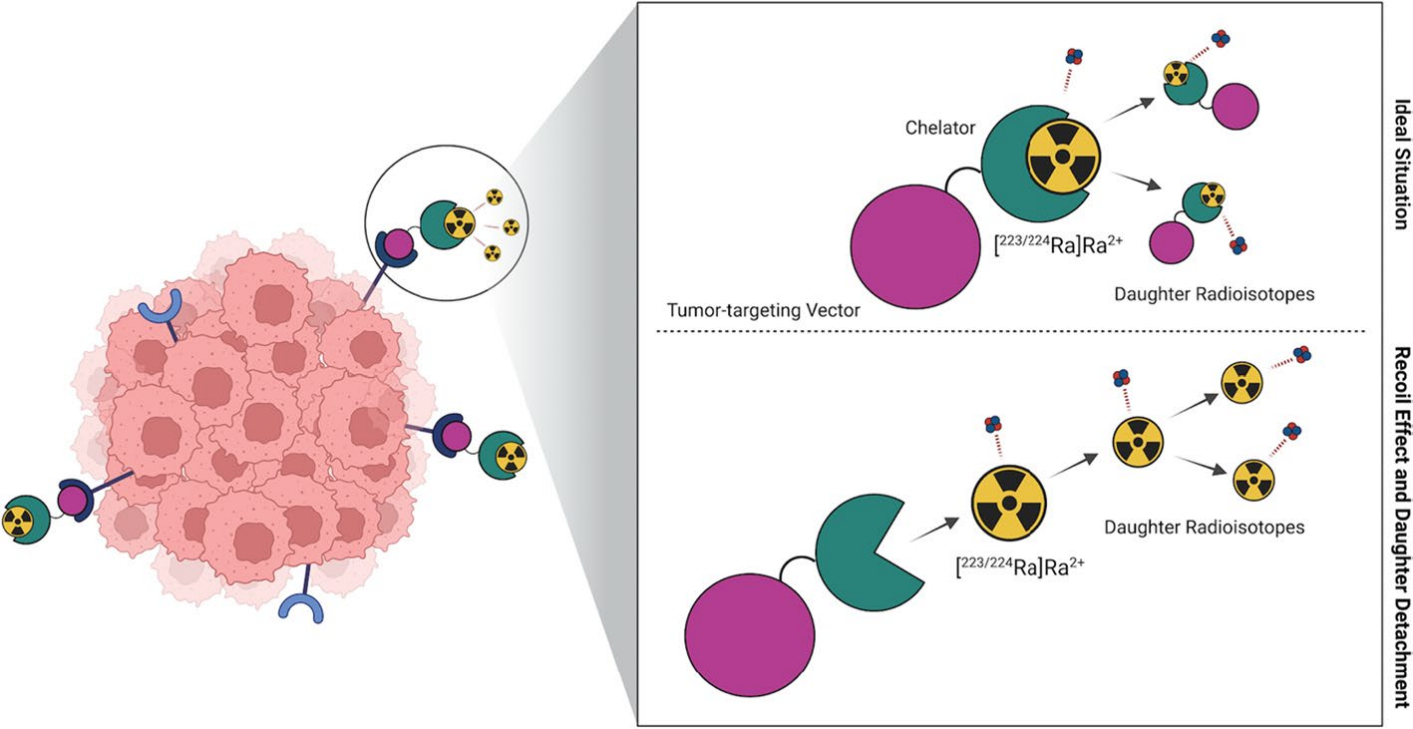
Representation of the bifunctional chelator approach in TAT: the ideal situation, in which the daughter radionuclides are retained by the chelator (upper), and the most probable situation, where the recoil effect causes the release of daughter radionuclides (lower). Image created with https://www.biorender.com

When the BFC approach is applied to TAT an additional concern must be kept in consideration. In fact, the recoil energy released from a parent α-emitting radionuclide is, in most cases, remarkably higher than the energy of any chemical bond (> 100 keV *vs*. ~ 5 eV on average, respectively) (de Kruijff et al. 2015). The structure encapsulating the radiometal can thus be cleaved upon α-decay, and the newly formed daughter (radio)nuclides can be released. Thus, it is of utmost importance to be acquainted not only with the nature, chemistry, and biological distribution of the parent α-emitter but also of the daughter nuclides, which are often α or *β*^−^ emitters themselves. In fact, the unleashing and potential redistribution in the body of these radionuclides can lead to unwanted toxicity in non-target healthy compartments (Fig. 2) (Ferrier and Radchenko 2019; de Kruijff et al. 2015; Poty et al. 2018). Furthermore, it is unlikely that the daughters are rapidly trapped back by the BFC, both because it is not guaranteed that they have the same chemical behavior as the parent and because several biological ligands (*e.g.*, serum proteins) can compete for their binding (de Kruijff et al. 2015).

The relevance of the recoiling daughters and their redistribution depends on numerous factors: (*i*) the individual half-life (*t*_1/2_) of the daughter radionuclide– the longer the *t*_1/2_ the more chances exist to cause undesired damage, (*ii*) the chemical nature of the daughter, (*iii*) passive transport phenomena, which can enhance the diffusion in certain tissues and organs (*e.g.*, tumors or kidneys) and depend on the medium (*e.g.*, blood, extra or intracellular matrix), (*iv*) active transport phenomena, such as carrier proteins and cell membrane chaperons, and (*v*) the intrinsic affinity of the daughter radionuclide for certain organs (de Kruijff et al. 2015; Poty et al. 2018).

Although hundreds of radionuclides decay by α-emission, only a few of them are so far considered suitable for TAT applications and several characteristics must be deeply considered when their use is practically evaluated for nuclear medicine purposes. Firstly, the radionuclide should be promptly available to hospitals. This means that it should be produced in sufficient amounts for the treatments and with a radionuclidic purity and molar activity suitable for medical applications (Mikołajczak et al. 2019; Ramogida and Orvig 2013). Favorable decay properties are equally important, *i.e.* the desired emission should have an energy suitable for the application, a high branching ratio, and a high percent abundance (Ferrier and Radchenko 2019; Ramogida and Orvig 2013). Secondly, the *t*_1/2_ of the radionuclide should fit the desired purpose: it should be neither too long, to avoid unnecessary and unwanted exposure of the patient, nor too short, to guarantee sufficient time for production, chemical processing, and shipping. Finally, the whole decay chain, including the daughter radionuclides, has to be assessed in order to spare potential redistribution in the body. That being stated, the main α-emitters currently considered for TAT are actinium-225 (^225^Ac, *t*_1/2_ = 9.9 d), bismuth-212 (^212^Bi, *t*_1/2_ = 60.6 min), bismuth-213 (^213^Bi, *t*_1/2_ = 45.6 min), thorium-226 (^226^Th, *t*_1/2_ = 30.6 min), thorium-227 (^227^Th, *t*_1/2_ = 18.7 d), astatine-211 (^211^At, *t*_1/2_ = 7.2 h), terbium-149 (^149^Tb, *t*_1/2_ = 4.1 h), radium-223 (^223^Ra, *t*_1/2_ = 11.4 d) and radium-224 (^224^Ra, *t*_1/2_ = 3.6 d) (Ferrier et al. 2019; Ferrier and Radchenko 2019; Yang et al. 2022).

This review focuses on the role of radium isotopes in nuclear medicine, from their decay properties and production to their biological and medical application, with particular attention to ^223^Ra. The aim of this work is to summarize the medical applications of radium and highlight the ongoing research lines towards its challenging but very appealing employment for TAT of various cancer types.

## Radium radioisotopes in nuclear medicine: decay properties and production routes

**Fig. 3:**
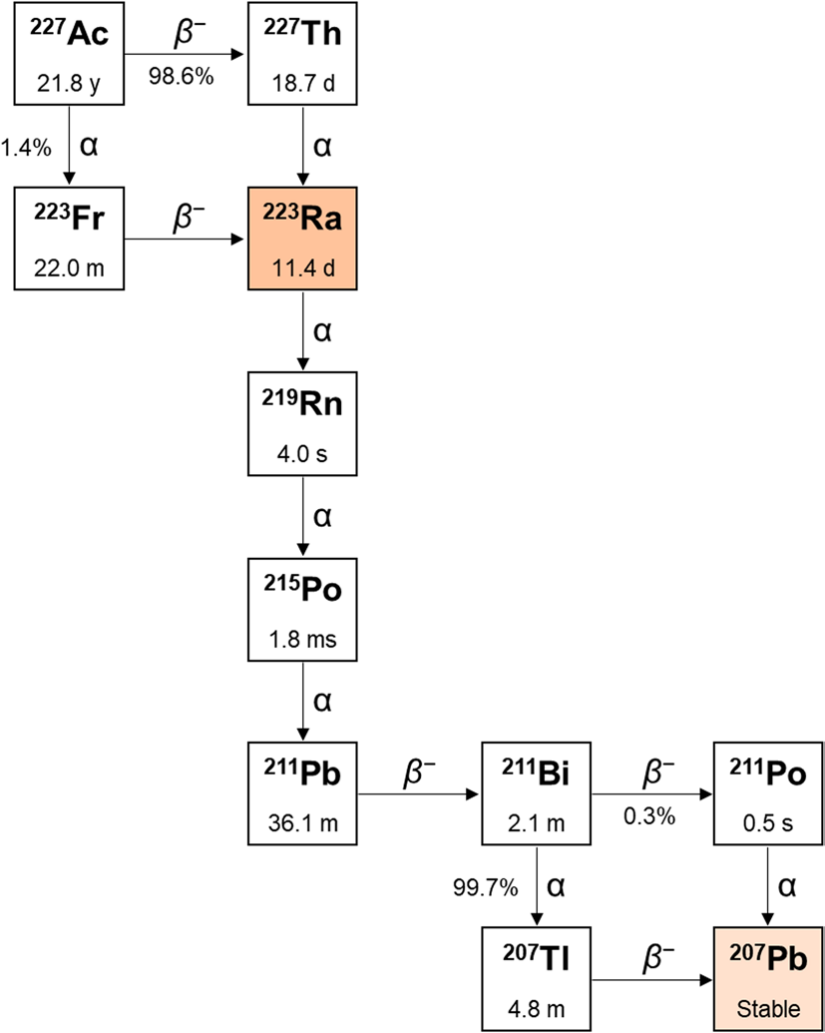
Origin and decay chain of ^223^Ra

**Fig. 4:**
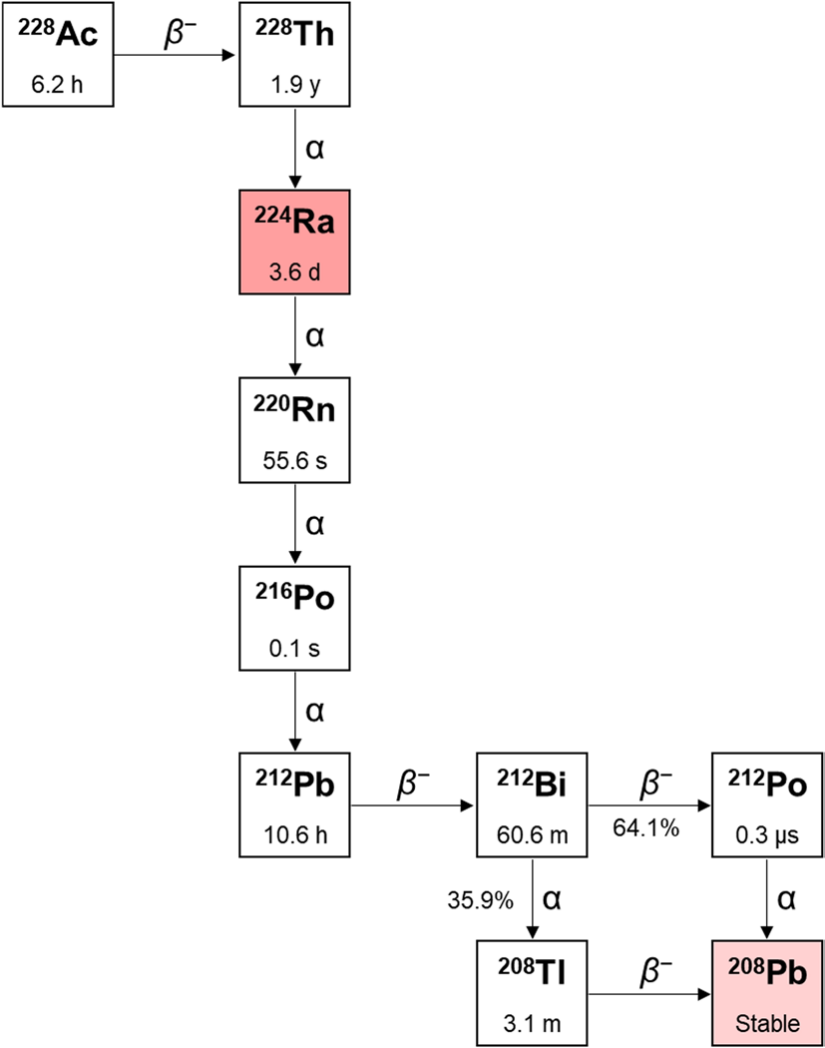
Origin and decay chain of ^224^Ra

All the 33 isotopes of radium are radioactive and four of them are naturally occurring from the decay of primordial nuclides, *i.e.* thorium-232 (^232^Th), uranium-235 (^235^U), and uranium-238 (^238^U) (Bauer et al. 2018; Ferrier and Radchenko 2019). Among these, radium-226 (^226^Ra, α-emitter, *t*_1/2_ = 1600 y) and radium-228 (^228^Ra, *β*^−^-emitter, *t*_1/2_ = 5.7 y) have half-lives which are too long to be meaningful for radionuclide therapy applications (Jia and Jia 2012). On the other hand, radium-223 (^223^Ra, *t*_1/2_ = 11.4 d) and radium-224 (^224^Ra, *t*_1/2_ = 3.6 d) decay exclusively via α-emission to radon-219 (^219^Rn, *t*_1/2_ = 4.0 s) and radon-220 (^220^Rn, *t*_1/2_ = 55.6 s), respectively, and have suitable half-lives for α-particle therapy. Additionally, their relatively long half-life allows the delivery to practically any locations devoted to patient treatment (Wadas et al. 2014). Complete ^223^Ra and ^224^Ra decay chains are depicted in Figs. 3 and 4, respectively (Ferrier and Radchenko 2019; IAEA; Radchenko et al. 2021).

For both radionuclides, the most abundant emitted α-particles are released with an energy (*E*_α_) of ~ 5.7 MeV (*E*_α_ = 5.72 MeV, *I* = 51.2% for ^223^Ra; *E*_α_ = 5.69 MeV, *I* = 94.9% for ^224^Ra) (Bauer et al. 2018; Ferrier and Radchenko 2019; IAEA; Poty et al. 2018). Both of them exhibit similar decay chains passing through a series of six daughters and emitting a total of four α- and two *β*^−^-particles before reaching a stable lead isotope (*i.e.* lead-207 – ^207^Pb – for ^223^Ra and lead-208 – ^208^Pb – for ^224^Ra). Both decay chains produce a comparable massive total energy release (28 MeV for ^223^Ra and 27 MeV for ^224^Ra) (Bauer et al. 2018; Ferrier and Radchenko 2019). The high energy emission, mainly due to the release of short-ranged α-particles, makes radium radioisotopes very potent therapeutic tools but also represents a major drawback for their stable incorporation in biological vectors due to the recoil effect (Herrero Álvarez et al. 2021; Huclier-Markai et al. 2012; Morris et al. 2019).

### ^223^Ra

The fraction of energy emitted by ^223^Ra and its daughters as α-particles is 95.3% but its overall decay is also accompanied by the release of *β*^−^-particles (3.6% energy) and γ-radiation (1.1% energy) (Fig. 3) (Bayer 2022; Poeppel et al. 2018). In particular, the 269 keV γ-emission (13.3% abundance) may be exploited to assess the biodistribution and pharmacokinetic of ^223^Ra-based radiopharmaceuticals via single-photon emission computed tomography (SPECT) imaging although the images’ quality is very low (Ferrier et al. 2019; Hindorf et al. 2012; IAEA; Poty et al. 2018).

While other therapeutic α-emitters, such as ^225^Ac and ^213^Bi, are currently produced only in quantities adequate for preclinical and relatively small clinical trials and are not able to meet the demands of global therapeutic applications in medicine, ^223^Ra is readily available worldwide and a shortage is not expected (Herrero Álvarez et al. 2021; Makvandi et al. 2018).

^223^Ra is currently obtained from the decay of ^227^Th, which is in turn produced from actinium-227 (^227^Ac, *t*_1/2_ = 21.8 y) in ^227^Ac/^227^Th generators (Fig. 3) (Ferrier and Radchenko 2019; Poty et al. 2018; Radchenko et al. 2021).

The production of ^227^Ac was recently reviewed by Radchenko et al*.*, and it is primarily achieved via neutron irradiation of ^226^Ra-enriched targets in nuclear reactors (Ferrier et al. 2019; Radchenko et al. 2021). Alternative routes of production include the recovery of ^227^Ac from legacy actinium-beryllium neutron sources and from the accelerator-based production of ^225^Ac which generates small quantities of ^227^Ac as by-product as well (Ferrier and Radchenko 2019; Radchenko et al. 2021). The main production of ^223^Ra is nowadays provided by Bayer HealthCare Pharmaceuticals Inc. and it is commercialized as an isotonic [^223^Ra]RaCl_2_ solution under the brand name Xofigo^®^ (formerly Alpharadin^®^).

### ^224^Ra

The decay chain of ^224^Ra includes α- and *β*^−^-emissions and, in addition, it is accompanied by the release of a 241 keV γ-ray of 4.1% abundance (Fig. 4) (Ferrier et al. 2019; IAEA; Poty et al. 2018).

^224^Ra can be obtained from the decay of thorium-228 (^228^Th, *t*_1/2_ = 1.9 y) which is in turn a decay daughter of both the primordial thorium-232 (*t*_1/2_ = 1.4·10^10^ y) and the long-lived uranium-232 (^232^U, *t*_1/2_ = 68.9 y) (Ferrier and Radchenko 2019; Kotovskii et al. 2015; Radchenko et al. 2021). ^228^Th can also be produced via neutron irradiation of ^226^Ra, resulting in ^228^Ra which decays in two successive *β*^−^ steps to actinium-228 (^228^Ac, *t*_1/2_ = 6.1 h) and finally to ^228^Th through the nuclear reactions ^226^Ra(2n,γ)^228^Ra(*β*^−^) → ^228^Ac(*β*^−^) → ^228^Th (Ferrier et al. 2019; Ferrier and Radchenko 2019; de Kruijff et al. 2015; Radchenko et al. 2021).

## ^223^Ra- and ^224^Ra-chloride for alpha particle therapy of bone diseases

**Fig. 5:**
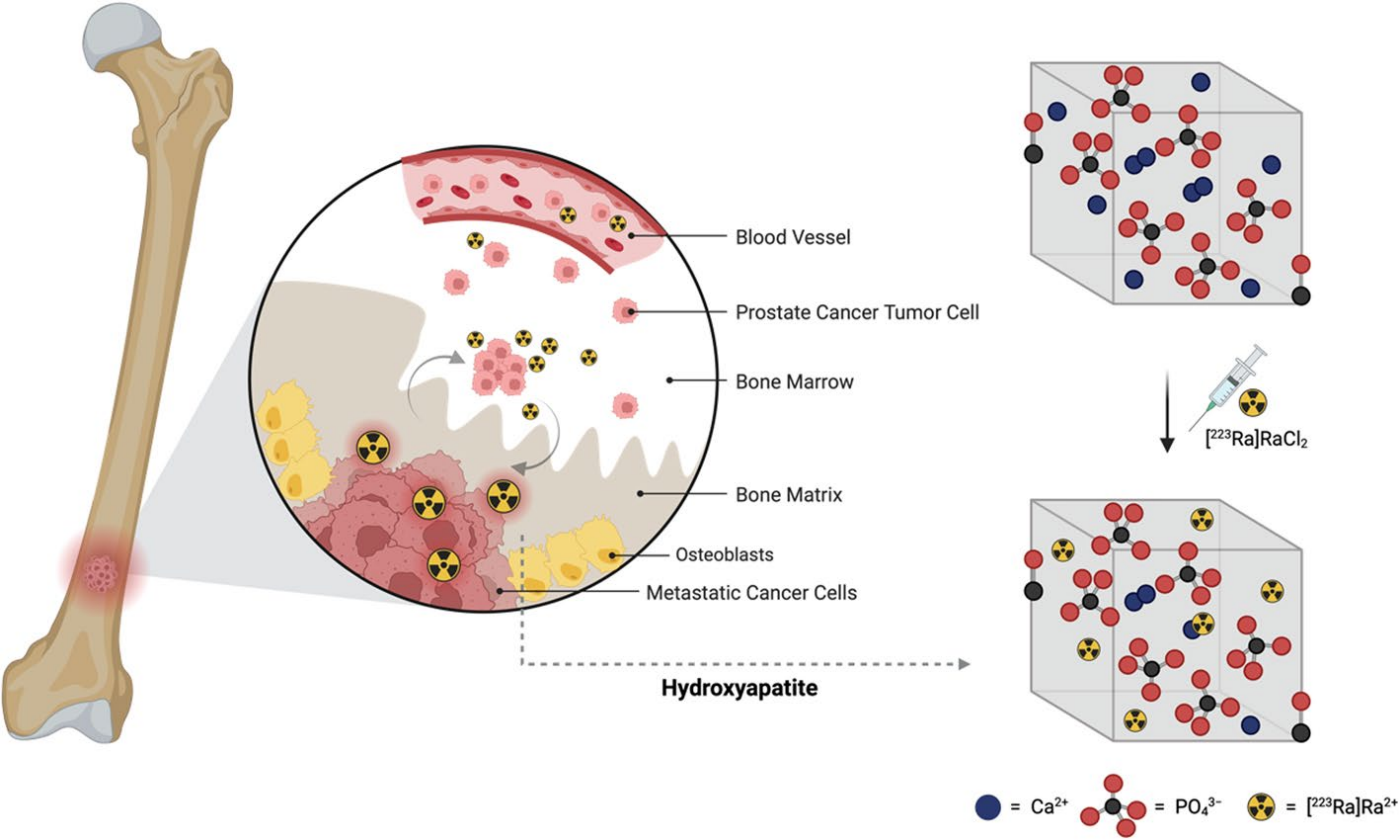
Representation of the distribution of ^223^Ra in the bone when injected as [^223^Ra]RaCl_2_. Image created with https://www.biorender.com

The cationic form of radium (Ra^2+^) possesses physiological similarities with calcium (Ca^2+^) as it occupies vacancies in the crystal lattice of hydroxyapatite, *i.e.* the mineral constituting the inorganic bone matrix. Ra^2+^ is thus an intrinsic “bone seeker” and it selectively accumulates in the bone, mainly in areas of high bone turnover like the border zones and the bone metastases (Fig. 5) (Poeppel et al. 2018). Thanks to its intrinsic biological properties, in 1903, Alexander Graham Bell suggested the use of radium for tumor therapy (Bell 1903).

### ^223^Ra

Advanced prostate cancer commonly gives rise to skeletal metastases that often cause bone pain, pathologic fracture, or spinal cord compression, which result in morbidity and mortality and necessitate treatment. Given the bone-seeking nature of radium cation, the energy released by [^223^Ra]Ra^2+^ is employed to produce a palliative anti-tumor effect on bone metastases. This therapeutic effect is ascribed to the multiple α-particles emitted that induce cellular damage on the osteoblastic prostate cancer metastases due to irreversible DNA breaks (Brito and Etchebehere 2019).

Preclinical and clinical experiments on this use of [^223^Ra]Ra^2+^ have been already extensively reviewed by Sgouros et al. (2010). As an example, Henriksen et al. (2002a) studied the effects of [^223^Ra]RaCl_2_ in nude rats to determine the therapeutic efficacy in a bone metastases model. The authors found that [^223^Ra]Ra^2+^ exhibited a marked anti-tumor effect at all the administered activities (60–110 kBq/kg). The radiotracer was well-tolerated by red marrow and no significant bone loss was detected (Henriksen et al. 2002a). In 2013, [^223^Ra]RaCl_2_ received the approval from the U.S. Food and Drug Administration (FDA) and the European Medicines Agency (EMA) for the treatment of adult patients with castration-resistant prostate cancer with symptomatic bone metastases and no known visceral metastatic disease (Bauer et al. 2018; Makvandi et al. 2018; Poeppel et al. 2018). [^223^Ra]RaCl_2_ (Xofigo^®^) is thus the first and, until now, the only α-particle emitting radiopharmaceutical approved for clinical use. Evidence of significant benefit, both in terms of overall survival and time to the first symptomatic skeletal-related event, has been associated with the treatment with Xofigo^®^. The procedure consists of repeated intravenous administration of saline solutions of [^223^Ra]RaCl_2_ in citrate buffer (6 injections at 4-week intervals, 55 kBq *per* kg body weight, *i.e.* usually < 8 MBq per injection) (Poeppel et al. 2018). [^223^Ra]Ra^2+^ is rapidly cleared from the blood (after 15 min from the injection, 80% is eliminated from the vascular bed and less than 1% remains in the blood at 24 h) and distributed into bone and bone metastases (Nguyen et al. 2016). A significant amount of activity is also excreted through intestine and feces, which represent the principal elimination pathway. At 7 days after injection, 80% of the administered activity is cleared from the body while the remaining part is embedded into the skeleton (Herrero Álvarez et al. 2021; Nguyen et al. 2016). Since its clearance does not depend on renal function, only 5% of [^223^Ra]Ra^2+^ is eliminated through urine, and no biliary excretion occurs, Xofigo^®^ is not considered nephrotoxic or hepatotoxic and this is a clear advantage over other therapies (Brito and Etchebehere 2019). On the other hand, treatments with radiopharmaceuticals may potentially increase the risk of contracting secondary cancer, in particular osteosarcoma, myelodysplastic syndrome, and leukemia, since they enhance the cumulative radiation to which a patient is exposed. However, differently from what is reported about [^224^Ra]RaCl_2_ (vide infra), no cases of cancers induced by ^223^Ra-based treatments have been observed in clinical trials during follow-up (Bayer 2022; Poeppel et al. 2018).

Combination of [^223^Ra]RaCl_2_ with chemotherapeutics such as Docetaxel or Cebazitaxel or with hormonal therapies such as Abiraterone and Enzalutamide have been and are currently being investigated in order to improve survival and decrease bone-related morbidity (*e.g.*, NCT03305224) (Cursano et al. 2020; Herrero Álvarez et al. 2021). However, contrasting outcomes have been reported as recently critically reviewed by Cursano et al. (2020). For example, in a randomized phase III trial when treatment of castration-resistant prostate cancer with Abiraterone was compared to Abiraterone + [^223^Ra]RaCl_2_, improvement of symptomatic skeletal event-free survival was not found (Smith et al. 2019). Moreover, an increased incidence of fractures and increased risk of radiological non-bone progression have been detected (*e.g.*, NCT02043678) (Herrero Álvarez et al. 2021). On the other hand, in a phase I study using a combination of Olapaib and [^223^Ra]RaCl_2_ in men with metastatic castration-resistant prostate cancer with bone metastases, early clinical benefit was observed and the combination will be further investigated in a phase II study (Pan et al. 2023).

Due to its undeniable clinical relevance in advanced metastatic prostate cancer, the use of [^223^Ra]RaCl_2_ has been also investigated (and is currently investigated) in other bone-metastatic tumors such as advanced breast cancer, advanced renal cell carcinoma and advanced thyroid cancer (Coleman et al. 2014; Deandreis et al. 2019; McKay et al. 2018; Nilsson et al. 2005). For example, in a phase IB/IIA clinical trial, the safety, feasibility and efficacy of the combination of [^223^Ra]RaCl_2_ (55 kBq/kg day 1 given on 6 weekly schedule) with Capecitabine chemotherapy (1000 mg/m^2^, twice daily, days 4–17 every 21 days) have been evaluated in patients with metastatic breast cancer (Winter et al. 2022). The mixed treatment was considered safe and feasible at the planned dose but no efficacy signals were seen (Winter et al. 2022). On the other hand, in another phase II clinical trial (NCT02366130), patients received intravenous injections of [^223^Ra]RaCl_2_ (55 kBq/kg) every 4 weeks up to 6 cycles together with endocrine therapy. The results demonstrated that [^223^Ra]RaCl_2_ plus hormonal therapy shows possible efficacy in hormone receptor-positive bone-dominant metastatic breast cancer and adverse events were tolerable (de Kruijff et al. 2015; Ueno et al. 2020; Winter et al. 2022).

### ^224^Ra

^224^Ra, historically known as Thorium-X, was initially introduced in medicine as a cure for benign dermatoses and vascular lesions, as well as sciatica, articular rheumatism, and secondary anemia (Ferrier et al. 2019). Subsequently, ^224^Ra chloride ([^224^Ra]RaCl_2_) was used to treat patients with non-cancerous bone diseases, in particular ankylosing spondylitis – a chronic and progressive inflammatory disease of the axial skeleton (Juzeniene et al. 2018; Poty et al. 2018; Priest et al. 2020). However, this option was discontinued in 2005 due to the incidence of leukemia and other tumors in patients, and ^224^Ra has not been employed in clinical settings anymore (Juzeniene et al. 2018; de Kruijff et al. 2015; Priest et al. 2020). Nowadays, only a few studies are reported about the revival and possible uses of [^224^Ra]RaCl_2_ for cancer treatment (Juzeniene et al. 2018). One example is the preclinical evaluation of an aqueous solution containing two bone-seeking agents to reduce breast cancer bone metastases in a mouse model (Juzeniene et al. 2018; de Kruijff et al. 2015). The components of the solution are [^224^Ra]RaCl_2_ and ethylenediaminetetra(methylene) phosphonic acid (EDTMP), which is used as a potential chelator of the daughter ^212^Pb with the aim to exploit it as an in vivo generator of ^212^Bi.

## Radium radioisotopes for targeted alpha particle therapy of non-osseous cancers

The clinical success of Xofigo^®^ motivates a high interest to advance the use of ^223^Ra also toward non-osseous cancers using a targeted approach. To this purpose, [^223^Ra]Ra^2+^ has to be ensnared in a carrier system able to vehicle it to the target tissue and overcome its spontaneous tendency to accumulate in the bones. The BFC approach is usually the most employed system to build up such a kind of metal-based targeted radiopharmaceuticals. However, this pathway has been scarcely investigated for radium so far, likely because the complexation chemistry of this element is still quite unexplored (Brown et al. 2022; Ivanov et al. 2022; Nelson et al. 2021). Other possible approaches such as encapsulation of ^223^Ra (and ^224^Ra) in nanocarriers and localized administration in tumor sites have been proposed and are under investigation. Indeed, these approaches might allow the targeted delivery of α-radiation to specific tissue and, at the same time, prevent the uncontrolled redistribution of recoiling daughters (de Kruijff et al. 2015). In the following paragraphs, these two approaches are reviewed.

### Towards the chelation of radium radioisotopes

A BFC suitable to complex a radionuclide as a part of a radiopharmaceutical should ideally meet a series of stringent requirements. Firstly, the metal-chelator complex should possess high thermodynamic stability and kinetic inertness in vivo to avoid the release of the radiometal in the bloodstream and prevent its biodistribution according to its intrinsic biological behavior (Bauer et al. 2018; Boros and Packard 2019; Price and Orvig 2014; Randhawa et al. 2021). This means that the complex should withstand transchelation phenomena by endogenous competing agents (*e.g.*, serum proteins and biological chelators) and remain intact at the extremely dilute conditions faced after the injection (*i.e.* nanograms of radiotracer diluted in the bloodstream). These characteristics are more commonly attained when rigid and structurally pre-organized macrocyclic chelators are employed (Franchi et al. 2022; Kostelnik and Orvig 2019). Secondly, the BFC should be selective for the radiometal of interest at trace levels, being able to compete against much higher amounts of potential non-radioactive impurities that could cause the impossibility of an efficient binding. Fast complexation kinetics and high radiometal incorporation under mild conditions (*i.e.* ambient temperature and neutral pH) are usually preferred, and become mandatory when temperature- or pH-sensitive biological vectors (like antibodies) are employed. These last features are generally favored by the more flexible open-chain acyclic chelators (Franchi et al. 2022; Kostelnik and Orvig 2019).

Comprehending the basic coordination chemistry of radium ions is therefore essential to build chemically tailored chelators for TAT of pathologies other than bone cancer (Boros and Packard 2019; Price and Orvig 2014). A concise overview of the aqueous chemistry of Ra^2+^ is given before delving into the various BFC approaches explored so far with radium radioisotopes.

#### Aqueous chemistry of radium

Radium is the heaviest element of the alkaline earth metals (Bauer et al. 2018; Curie 1911). Since all its isotopes are radioactive and emit high-intensity radiations, it is difficult to work with milligram amounts and millimolar concentrations that are typically required to obtain coordination chemistry data (*i.e.* thermodynamic stability, formation and dissociation kinetics, structural properties). Therefore, the knowledge about the aqueous chemistry of radium is quite limited and it is often based on the analogies between this element and other alkaline earth metals, especially barium (Brown et al. 2022; Curie 1911; Gott et al. 2016).

Like barium and the other elements belonging to group II, radium is present in aqueous solutions exclusively in the + 2 oxidation state as a hydrated cation (Gott et al. 2016; Grieve and Paterson 2022; Yamaguchi et al. 2022). The chemistry of barium and radium is similar, yet the small difference in their ionic radius (142 pm for Ba^2+^ *vs.* 148 pm for Ra^2+^, referred to coordination number 8) (Shannon 1976) is still sufficient to cause measurable differences in the solubility of their compounds and in the stability of their complexes with the same ligand(s). Radium exhibits a highly basic character and is therefore difficult to complex (vide infra), so most of its known compounds are simple ionic salts (Gott et al. 2016).

Generally, Ra^2+^ compounds are less soluble in water than the Ba^2+^ analogues, although in some cases the opposite has been observed (Brown et al. 2022). As an example of these exceptions, Ra(OH)_2_ is the most soluble of all the alkaline earth hydroxides and is more basic than Ba(OH)_2_. As well, RaCO_3_ is reported to be more soluble than the barium analogue, probably due to a highly disordered crystalline structure, as confirmed by a very recent study (Brown et al. 2022; Matyskin et al. 2023). While RaSO_4_ and RaHPO_4_ have low solubility products, RaCl_2_ and RaBr_2_ are relatively soluble, with the bromide being more soluble than the chloride (Brown et al. 2022). These two halides are the only commercial compounds of radium. Though radium salts are fairly stable to radiolysis, they degrade and darken over time as a result of radiation damage (Gott et al. 2016).

The first single crystal X-ray diffraction characterization of a pure Ra^2+^ compound was only very recently published by Bai et al. (2023). In the crystal structure, Ra^2+^ interacts with six nitrate anions, each lending two oxygen atoms, and results in a 12-coordinated [Ra(NO_3_)_6_]^4−^ environment best described as an anticuboctahedron (Johnson solid *J*_27_) with slight distortions from a regular prism (Bai et al. 2023). Ra(NO_3_)_2_ is isomorphous with Ca(NO_3_)_2_, Sr(NO_3_)_2_, Ba(NO_3_)_2_, and Pb(NO_3_)_2_ but the metal-nitrate bonds become weaker with increasing ionic radius. As expected, in Ra(NO_3_)_2_, the Ra-O interactions are predominantly electrostatic but are stabilized by donation from the lone pairs of O atoms to the 7*s* orbital of Ra^2+^ (~ 5 kcal mol^−1^). On the contrary, the Ra-nitrate orbital mixing is negligible (Bai et al. 2023).

#### Coordination chemistry of radium

Due to the large electronegativity difference between the metals and the donor atoms, metal–ligand interactions are considered predominantly ionic for alkaline earth metals (Fromm 2020). As a consequence, these interactions are non-directional and are mostly governed by electrostatic and steric factors. However, evidence of good solubilities in organic solvents and the presence of unusual geometries suggest a certain grade of covalency in some alkaline earth metal compounds (Fromm 2020). Additionally, the coordination chemistry of group II metals is challenging because closed-shell metal ions do not show a preference for specific coordination geometries. The chemical behaviour of radium seems similar to that of barium, with Ra^2+^ complexes being generally weaker than the Ba^2+^ analogues, but the coordination chemistry of radium has been scarcely explored so far (Brown et al. 2022; Fromm 2020) and the aquo ion and the hydration properties of Ra^2+^ have only been studied and rationalized very recently. Yamaguchi et al*.* reported a coordination number of 9.2 ± 1.9 for [Ra(H_2_O)_n_]^2+^ and an average distance between the radium ion and the oxygen atoms in the first hydration shell of 2.87 ± 0.06 Å. Among the alkaline earth cations, Ra^2+^ has the less structured water molecules in the first hydration shell, making the aquo ion significantly labile (Yamaguchi et al. 2022). Water lability in Ra^2+^ complexes is also supported by the single-crystal X-ray diffraction structure of a molecular compound of Ra^2+^ with dibenzo-30-crown-10 (DB30C10), which shows that Ra-O_water_ bond is much longer than Ba-O_water_ in similar solid-state structures (White et al. 2023).

**Fig. 6:**
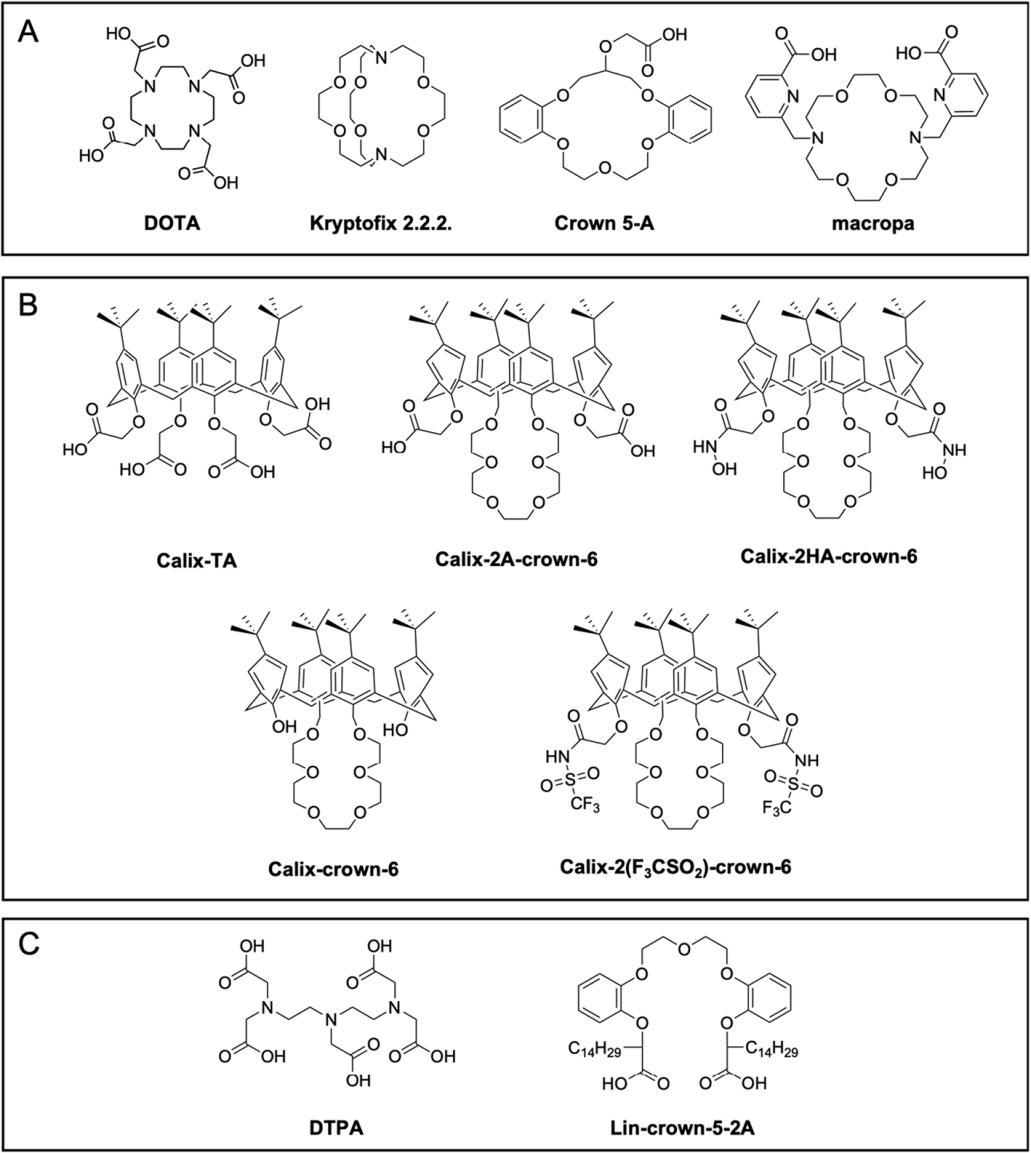
Structures of **A** macrocyclic, **B** supramolecular, and **C** acyclic chelators proposed for ^223^Ra-complexation

The weak complex [Ra(OH)]^+^ has been observed experimentally (log*K*^0^ = 0.57, where the symbol *K*^0^ refers to the stability constant *K* corrected to zero ionic strength) and stability constants have also been estimated for [RaCl]^+^ (log*K*^0^ =  − 0.32) and [RaF]^+^ (log*K*^0^ =  − 0.27) but the latter complexes are unstable and the formation of [RaCl]^+^ may actually be questionable at all (Brown et al. 2022). The stability of several small organic molecules’ complexes with the heaviest alkaline earth metals (Sr^2+^, Ba^2+^, Ra^2+^) has been investigated by different authors and was collected in a review by Brown et al. (2022). The report includes some polyaminopolycarboxylic acids derivatives, such as ethylenediaminetetraacetic acid (EDTA), cyclohexane-1,2-diaminetetraacetic acid (CyDTA), diethylenetriaminepentaacetic acid (DTPA, Fig. 6), nitrilotriacetic acid (NTA), 2,2′-ethylenedioxyl bis[ethyl-iminodi(acetic acid)] (EGTA), N′-(2-hydroxyethyl)ethylenediamine N,N,N′-triacetic acid (HEDTA), and other organic acids derivatives such as citric, tartaric, malic, succinic, aspartic, pyruvic, oxaloacetic, fumaric, and sulfosalicylic acid (Brown et al. 2022). Indeed, “hard” donors (according to Pearson’s Hard and Soft Acids and Bases theory – HSAB (Pearson 1968)) like carboxylate, hydroxy-, and oxo- groups are expected to provide the strongest binding of alkaline earth cations (Henriksen et al. 2002b). All the considered complexes have a 1:1 metal-to-ligand stoichiometry with the stability of Ra-complexes depending on the ligand’s charge, as more negatively charged ligands give more stable complexes. The values of log*K*^0^ of neutral complex species are similar for organic and inorganic ligands, and they are generally lower for Ra^2+^ than for Ba^2+^. The set of stability constants of Ra^2+^ with the considered ligands shows a very good linear correlation with Ba^2+^, while a similar relationship does not exist with Sr^2+^. This consideration was found to be valid both when log*K*^0^ decreases in the series Sr^2+^ > Ba^2+^ > Ra^2+^ and when it increases as well. This finding is considered useful to estimate the stability constants that have not yet been measured experimentally such as, typically, the Ra^2+^ ones (Brown et al. 2022). In fact, as already outlined before, classical and established techniques for the analysis of complexation equilibria, like potentiometric and spectroscopic methods, are not feasible with Ra^2+^ due to their relatively high detection limits. Consequently, the stability of complexes with Ra^2+^ reported in the literature for radiopharmaceutical applications (mainly ^223^Ra-labelled complexes) was only determined indirectly through liquid–liquid extraction experiments and competition methods with respect to a selected ligand, coupled with γ-spectroscopy detection (Chen et al. 1999; Henriksen et al. 2002b). Alternatively, Ba^2+^ has been used as non-radioactive surrogate for stability constant calculation (Bauer et al. 2018; Steinberg et al. 2018). However, the small chemical differences between Ba^2+^ and Ra^2+^ are still sufficient to render these calculations an approximative estimation. Moreover, it is difficult to critically appraise the real potential of the chelators reported in the literature because, in most cases, they have not been studied more deeply than at a basic radiochemical level. Herein the acyclic, macrocyclic chelators, and supramolecular systems proposed for [^223^Ra]Ra^2+^ binding are summarized and critically analyzed.

##### Macrocyclic and supramolecular chelators

A few macrocyclic chelators have been proposed for ^223^Ra binding based on the considerations that the large ionic radius of Ra^2+^ may require a relatively high number of donor atoms and that a proper cavity size may provide selectivity over other metal cations (Henriksen et al. 2002b). Indeed, a high coordination number has been very recently observed in [Ra(DB30C10)(H_2_O)][BPh_4_]_2_ by single-crystal X-ray diffraction, where Ra^2+^ sits in a 11-coordinate environment bound to the ten crown-ether O atoms and one O from a hydration water molecule (White et al. 2023).

The proposed macrocyclic chelators include 1,4,7,10-tetraazacyclododecane-1,4,7,10-tetraacetic acid (DOTA) – one of the most frequently used chelators in nuclear medicine thanks to its ability to complex a wide variety of metal cations (Kostelnik and Orvig 2019)—, the bicyclic crown ether Kryptofix 2.2.2 (Kry 2.2.2), a crown ether monocarboxylic acid (Crown 5-A), and a 18-membered mixed crown ether azamacrocycle derivative (macropa). Moreover, supramolecular chelators belonging to the class of calixarenes or mixed calixarene-crowns were also assessed for [^223^Ra]Ra^2+^ complexation due to their acknowledged capability to complex alkaline earth metal ions (Bauer et al. 2018; Chen et al. 1999; Henriksen et al. 2002b; Steinberg et al. 2018). The structures of the macrocyclic and supramolecular chelators herein revised are reported in Fig. 6A, B.

DOTA and Kry 2.2.2 were inspected together with 4-*tert*-butylcalix[4]arene-tetraacetic acid (Calix-TA) by Henriksen et al. (2002b). Among the three, Calix-TA showed the highest affinity for Ra^2+^, its extraction constant in water/chloroform being 2.2- and 3.5-fold higher than DOTA and Kry 2.2.2., respectively (Henriksen et al. 2002b).

[Ra(DOTA)]^2−^ formation constant was only recently determined and resulted in a log*K* value of 7.82. This moderate stability was ascribed to the significant affinity of DOTA for Na^+^, which were part of the medium used for the measurements and can compete with Ra^2+^ in the complexation (Ivanov et al. 2022). In agreement with these findings, radiolabeling experiments with [^223^Ra]Ra^2+^ were recently conducted on DOTA but resulted in poor yields (vide infra) (Abou et al. 2021).

Since in the study by Henriksen et al. (2002b) the complex between [^223^Ra]Ra^2+^ and Calix-TA was found the most promising, this ligand also underwent competition experiments against serum-abundant metals. The pre-formed [^223^Ra]Ra^2+^ complex was extracted in chloroform and then mixed with an HEPES solution (pH 7.4) containing relevant metal cations (*i.e.*, Na^+^, K^+^, Mg^2+^, Ca^2+^, and Zn^2+^). Only 65% of [^223^Ra]Ra^2+^ activity remained in chloroform after 10 min of mixing, meaning that the complex with Calix-TA underwent rapid dissociation and transmetallation phenomena likely occurred. This result suggested that Calix-TA is not a suitable chelator for [^223^Ra]Ra^2+^ in vivo applications (Henriksen et al. 2002b).

Similar experiments were conducted by Chen et al*.* (1999) to compare a crown ether monocarboxylic acid (Crown-5-A) and two mixed calixarene-crowns (Calix-2A-crown-6 and Calix-2HA-crown-6). Crown-5-A showed limited ability to extract [^223^Ra]Ra^2+^ from water to chloroform and no selectivity over the other alkaline earth cations, therefore it was rapidly abandoned (Chen et al. 1999). On the other hand, the two mixed calixarene-crowns were rationally selected due to the following promising features: (*i*) the rigidity and cavity size of similar molecules were known to favor selectivity for Cs^+^ over K^+^, so it was hypothesized that they would be suitable for Ra^2+^ over smaller alkaline earth cations as well; (*ii*) neutral calixarene-crowns usually showed weak coordination with alkaline earth metal ions, thus the functionalization with proton-ionizable side-arm groups was expected to enhance complex stability; (*iii*) such scaffolds could be further easily bifunctionalized to allow the conjugation with a tumor-targeting vector (Chen et al. 1999).

Both calixarene-crown derivatives could quantitatively (> 99.9%) extract [^223^Ra]Ra^2+^ from water (8.0 < pH < 9.5) to chloroform. When competing with EDTA, Calix-2HA-crown-6 exhibited an extraction constant of around one order of magnitude higher than Calix-2A-crown-6. This outcome was attributed to the stronger acidity of the hydroxamic than the carboxylic acid group and to the lesser conformational change of Calix-2HA-crown-6 upon complexation compared to Calix-2A-crown-6 (Chen et al. 1999). The kinetic inertness of ^223^Ra-complexes with these two chelators was assessed in the presence of serum-abundant metal ions. The pre-formed complexes were extracted into chloroform and then underwent competition experiments with a pH 7.4 buffer solution containing Na^+^, K^+^, Mg^2+^, Ca^2+^, and Zn^2+^. After 24 h, only < 5% of [^223^Ra]Ra^2+^ was removed from the organic phase, pointing out the remarkable inertness of Ra-complexes with both calixarene-crown derivatives (Chen et al. 1999).

On these bases, the new derivative Calix-crown-6 was recently proposed as a possible leading compound for the chelation of heavy alkaline earth cations of radiopharmaceutical interest (*i.e.* Sr^2+^, Ba^2+^ and Ra^2+^) (Bauer et al. 2018). The study provided insight into the solution structure and stability constants of Calix-crown-6 complexes with Ba^2+^ and Sr^2+^, showing that they possess a 1:1 metal-to-ligand stoichiometry with log*K*_Ba_ = 4.7 and log*K*_Sr_ = 4.3, respectively (Bauer et al. 2018). In a subsequent study, the same researchers developed a series of calix-crown derivatives based on Calix-crown-6 bearing different functional groups on the two phenolic rings not bridged by the crown ether moiety (Steinberg et al. 2018). The stability constants of the complexes formed by these ligands with Ba^2+^ resulted in the same order of magnitude as the Calix-crown-6 one. Two derivatives in particular, *i.e.* the already mentioned Calix-2HA-crown-6 and Calix-2(F_3_CSO_2_)-crown-6 (Fig. 6B), exhibited a log*K* > 5 and appeared promising for further investigations, likely thanks to the electron-withdrawing groups which render them highly acidic and might stabilize the whole complex due to the formation of an ion pair with Ba^2+^ (Steinberg et al. 2018).

On the other hand, these two studies could not be considered indicative of the ability of Calix-crown-6 and its derivatives to complex [^223^Ra]Ra^2+^ under radiopharmaceutical relevant conditions (*i.e.* aqueous environment and extreme dilution), since they were only limited to thermodynamic measurements via spectroscopic methods in an organic solvent (Bauer et al. 2018; Steinberg et al. 2018). Moreover, the solubility of these complexes in aqueous media remains a concern for their therapeutic applications (Grieve and Paterson 2022).

The ligand macropa, a diaza-18-crown-6 ring bearing two picolinic acid pendants appended on the N atoms (Fig. 6A), has been considered for ^223^Ra-complexation (Abou et al. 2021; Roca-Sabio et al. 2009). Being based on an 18-membered macrocycle, which provides a spacious cavity, this ligand displays a preference for larger (such as La^3+^, Ce^3+^, Ac^3+^ and Ba^2+^) over smaller metal ions (Blei et al. 2023; Roca-Sabio et al. 2009; Thiele et al. 2017, 2018). Indeed, macropa has been successfully used to build up radiopharmaceuticals based on the matched theranostic pair lanthanum-132 (^132^La, *t*_1/2_ = 4.8 h, positron emitter)/lanthanum-135 (^135^La, *t*_1/2_ = 19.5 h, positron and Auger electron emitter) and ^225^Ac, upon conjugation with small molecules targeting prostate-specific membrane antigen (PSMA) (Aluicio-Sarduy et al. 2020; IAEA; Kelly et al. 2019; Thiele et al. 2017). Due to its ability to form Ba-complexes with high thermodynamic stability (log*K*_[Ba(macropa)]_ = 11.11), macropa was also suggested as potential chelator for Ra^2+^ (Abou et al. 2021; Thiele et al. 2018). According to density functional theory (DFT) calculations, Ra^2+^ in the [Ra(macropa)] complex should exhibit a tenfold coordination environment by interacting with the six donor atoms of the crown ring and with the two N and two O donors of the picolinate side arms, in analogy with the crystal structure determined for [Ba(Hmacropa)(DMF)]ClO_4_·Et_2_O (Ivanov et al. 2022; Thiele et al. 2018). A log*K*_[Ra(macropa)]_ = 10.00 was very recently measured via a cation exchange method, and the ligand attested to retain high capability to bind Ra^2+^ also under physiologically relevant conditions (pH 7.4 or Na^+^-rich ionic medium) (Ivanov et al. 2022).

Concentration-dependent ^223^Ra-labeling with macropa (pH 6) was performed by Abou et al*.*, and the chelator was able to incorporate [^223^Ra]Ra^2+^ (3.7 kBq) with high efficiency (> 80%) at 18 μM ligand concentration within 5 min at room temperature. Radiochemical purity greater than 95% was achieved for apparent molar activities ranging from 2.05 to 9.62 kBq/nmol. For comparison, DOTA (10 mM) and EDTA (5 mM) could complex only 15% and 35% of [^223^Ra]Ra^2+^, respectively (Abou et al. 2021). The integrity of [^223^Ra][Ra(macropa)] was probed by incubating the complex in human serum at 37 °C and ~ 90% of the compound was still intact after 12 days (Abou et al. 2021). Biodistribution in healthy, skeletally mature rodent models additionally pointed out the in vivo stability of this complex, as accumulation in the bone was one order of magnitude lower than that of [^223^Ra]RaCl_2_ (1.6% *vs*. 22% of injected activity, respectively) at 24 h post-injection. Both [^223^Ra][Ra(macropa)] and [^223^Ra]RaCl_2_ were rapidly cleared from the blood, but the former had a different excretion profile (*i.e.* renal *vs.* intestinal), with a higher accumulation in the bladder and lower uptake in the gut and spleen at 15 min post-injection. This might lead to an improvement in patient outcomes since gastrointestinal distress is a common symptom in patients treated with [^223^Ra]RaCl_2_. At 24 h most organs displayed < 0.01% of injected activity (Abou et al. 2021).

##### Acyclic chelators

The investigation of acyclic chelators for ^223^Ra-complexation is a rather unexplored field and includes the octadentate aminopolycarboxylic acid DTPA and a polyether dicarboxylic acid (Lin-crown-5-2A). The structures of these chelators are reported in Fig. 6C.

Among the relatively simple ligands collected in the review by Brown et al., DTPA – a widely used chelator for the complexation of medical relevant radionuclides (Okoye et al. 2019) – appeared the most promising one, forming a [^223^Ra][Ra(DTPA)]^3−^ complex with a quite remarkable stability (log*K*^0^ = 10.74) (Brown et al. 2022). DTPA was hence considered as a potential chelator for [^223^Ra]Ra^2+^ in vivo and was inspected in liquid–liquid extraction competition experiments with the already described [^223^Ra]Ra-Calix-TA (Brown et al. 2022; Henriksen et al. 2002b). A pre-formed lipophilic complex of [^223^Ra]Ra-Calix-TA in chloroform was mixed with an aqueous solution of DTPA to evaluate their relative performance. The amount of [^223^Ra]Ra^2+^ transferred into the water phase due to chelation by DTPA indicated an extraction constant 5.7-fold lower than Calix-TA (Henriksen et al. 2002b). The value was also lower than those reported for DOTA and Kry 2.2.2, suggesting that the absence of the macrocyclic effect is detrimental for the complex stability. After this finding, no further experiments have been conducted, to the best of our knowledge, for the evaluation of [^223^Ra]Ra-DTPA complexes.

Lin-crown-5-2A was another scaffold suggested as potential chelator for ^223^Ra-based radiopharmaceuticals (Chen et al. 1999). This ligand exhibited high extraction efficiency of [^223^Ra]Ra^2+^ from water to chloroform but, since it showed no selectivity for Ra^2+^ over the other alkaline earth metal cations (*i.e.* Mg^2+^, Ca^2+^, Sr^2+^, Ba^2+^), it was not further investigated (Chen et al. 1999).

### ***Preclinical tests for ***^***223***^***Ra-based TAT***

**Fig. 7:**
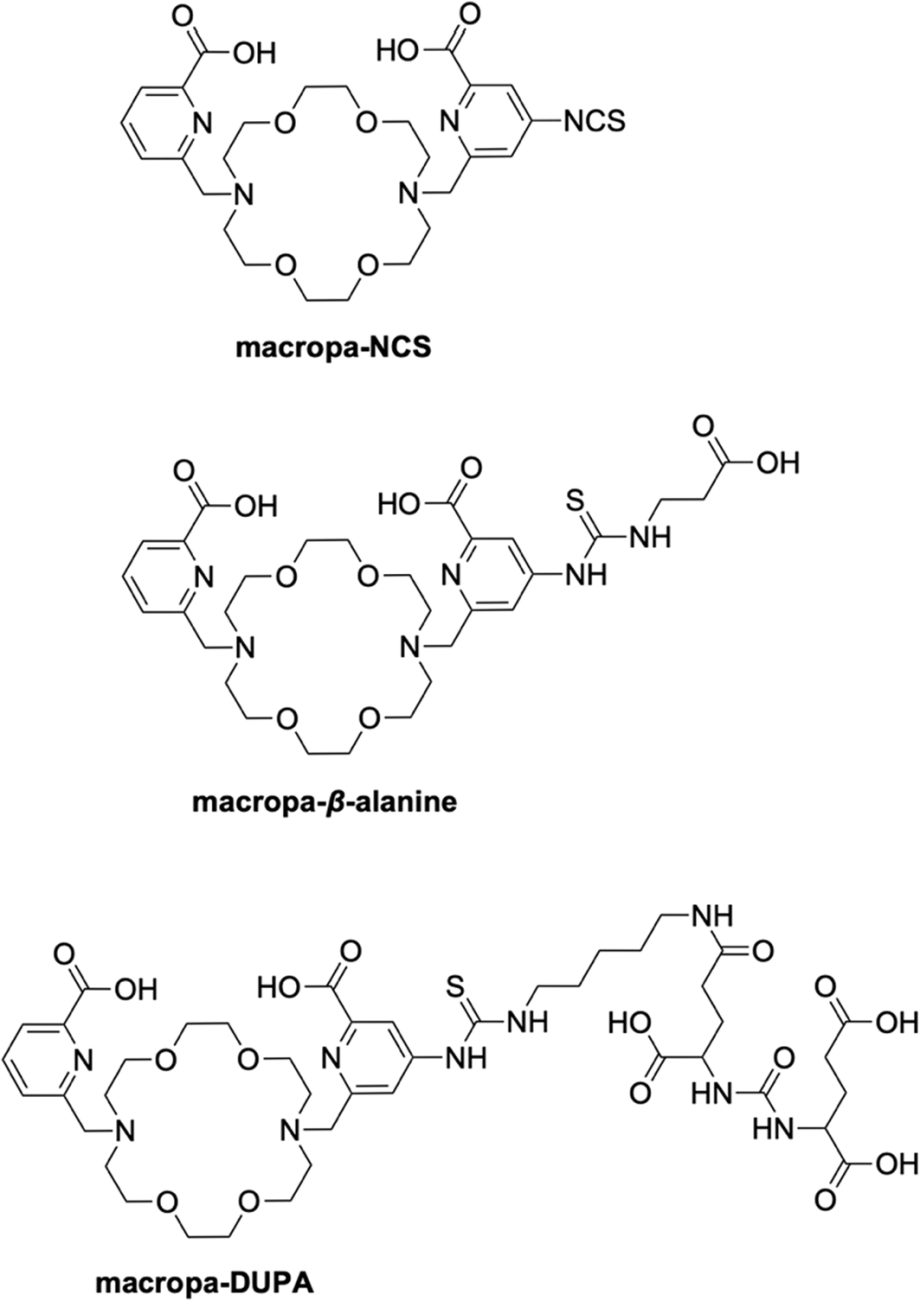
Structures of the bifunctional version of macropa (macropa-NCS), and its conjugates (macropa-*β*-alanine and macropa-DUPA)

Due to the promising results obtained with macropa, a bifunctional version of this chelator bearing an isothiocyanate moiety on one of the picolinic acid arms (macropa-NCS, Fig. 7) (Thiele et al. 2017) was conjugated either to the amino acid *β*-alanine or to the small molecule glutamate-urea-glutamate (DUPA), a targeting vector with high affinity to PSMA (Fig. 7) (Abou et al. 2021). Both constructs showed high labeling yield (> 90% for macropa-*β*-alanine, > 95% for macropa-DUPA) and fast incorporation (≤ 30 min) under mild conditions (room temperature, pH 6, 0.1 mM macropa conjugate, 3.7 kBq of ^223^Ra). Both complexes attested also to be stable in human serum as the intact fraction was > 70% for [^223^Ra][Ra(macropa-*β*-alanine)] and > 90% for [^223^Ra][Ra(macropa-DUPA)] after 12 d of incubation at 37 °C (Abou et al. 2021). When tested in murine models, [^223^Ra][Ra(macropa-*β*-alanine)] had a similar biodistribution to [^223^Ra][Ra(macropa)], with a lower bone, spleen, and kidney uptake and significantly lower activity in all tissues at 24 h post-injection compared to [^223^Ra]RaCl_2_. On the contrary, and quite unexpectedly, the biodistribution of [^223^Ra][Ra(macropa-DUPA)] was very similar to [^223^Ra]RaCl_2_, with high uptake in bone, spleen, and kidneys consistent with an in vivo release of [^223^Ra]Ra^2+^ from the conjugate.

Despite these results, the groundbreaking work of Abou et al*.* (Abou et al. 2021) demonstrated that the efficient complexation of [^223^Ra]Ra^2+^ under mild conditions and the preservation of remarkable stability both in human serum and in vivo can be achieved, thus demonstrating the feasibility of ^223^Ra-based TAT. However, much room still exists in the investigation of different conjugates that retain the favorable in vivo behavior of [^223^Ra][Ra(macropa)] or in the exploration of new chelating agents which are not negatively affected by the attachment to a biological targeting vector.

### Alternative approaches to bind radium radioisotopes

The development of new chelators able to prompt a bifunctional approach was mainly investigated for the delivery of [^223^Ra]Ra^2+^; to the best of our knowledge, [^224^Ra]Ra^2+^ has never been considered for TAT based on the BFC approach so far. Instead, other forms of tumor-targeted delivery have been recently explored for [^224^Ra]Ra^2+^, thus enriching the plethora of possible therapeutic approaches. These forms include the preclinical evaluation in mouse models of (*i*) ^224^Ra-loaded wires inserted into solid tumors (a form of brachytherapy called diffusing alpha-emitters radiation therapy – DART) for the treatment of prostate, glioblastoma, colon, squamous cell carcinoma and melanoma cancer cell, and (*ii*) calcium carbonate microparticles as ^224^Ra-carriers for the treatment of disseminated cancers such as those occurring in the peritoneum (Cooks et al. 2012; Nelson et al. 2021; Westrøm et al. 2018).

Another possibility that has been proposed in recent years to expand the utilization of Ra^2+^ for the treatment of non-osseous tumors is the incorporation of [^223/224^Ra]Ra^2+^ in nanocarriers. As opposed to the BFC approach, the use of nanocarriers does not strictly require a perfectly tailored chemistry (Kleynhans et al. 2021). Nanoparticles based on metal salts or oxides (Gaweda 2019; Reissig et al. 2019, 2020b; Rojas et al. 2015; Sakmár et al. 2023; Suchánková et al. 2020b, a) and nanozeolites have been considered (Czerwińska et al. 2020; Lankoff et al. 2021; Piotrowska et al. 2013, 2017). Indeed, these carriers displayed the capability to incorporate metal cations and retain both Ra radionuclides and their recoiling daughters. However, unwanted radioactivity accumulation in the spleen and liver has been reported in animal models treated with this approach, in turn leading to side effects due to irradiation of non-target organs (Lankoff et al. 2021; Reissig et al. 2021). Liposomal nanocarriers have also been considered but the accumulation in critical organs (*e.g.* spleen, liver, and lungs) is the main barrier toward their clinical translation (Kleynhans et al. 2021). Selected examples of nanocarriers that have been proposed in the literature, together with the most important outcomes from the collected studies, are summarized in Table 1.

**Table 1:** Summary of studies conducted with radium radioisotopes bound in nanocarriers

Nanocarrier	Incorporated radionuclide	Surface functionalization	Targeting vector	Radiochemical incorporation^[a]^	Stability^[b]^	In vitro and in vivo results	References
Barium sulphate (BaSO_4_) functionalized nanoparticles	^133^Ba ^[c]^	Alendronate	–	20% (single-step precipitation)	> 95% at 7 d in water	*Not explored*	Reissig et al. (2019)
	^224^Ra						
	^131^Ba ^[d]^	Alendronate	–	40% (two-step precipitation)	93% at 7 d in water	*Not explored*	Reissig et al. (2020b)
	^133^Ba			41% (two-step precipitation)			
	^224^Ra			31% (two-step precipitation)	96% (82% if measuring ^212^Pb) at 7 d in water		
Barium hexaferrite (BaFe_12_O_19_) functionalized nanoparticles	^223^Ra	3-phosphonopropionic acid (CEPA)	Trastuzumab	70% (autoclave method)	Almost quantitative in 0.01 M phosphate buffered saline (PBS), 0.9% NaCl, and human blood serum (20% release of ^211^Pb)	High affinity to human epidermal growth factor receptor 2 (HER2) on ovarian cancer cells High cytotoxicity	Gaweda (2019)
Titanium dioxide (TiO_2_) nanoparticles	^223^Ra	–	–	98.7% (surface sorption) 99.1% (single-step precipitation)	> 99% at 59 h and 55 d in saline; > 97.5% at 59 h and > 97% at 55 d in bovine blood serum	*Not explored*	Suchánková et al. (2020a)
Hydroxyapatite (Ca_10_(PO_4_)_6_(OH)_2_) nanoparticles	^223^Ra	–	–	94.2% (surface sorption) 97.0% (single-step precipitation)	~ 40% at 59 h and ~ 80% at 55 d in saline; ~ 78% at 59 h and > 80% at 55 d in bovine blood serum	*Not explored*	Suchánková et al. (2020a)
Lanthanum phosphate (LaPO_4_) nanoparticles	^223^Ra	–	–	91% (precipitation)	87.3% at 35 d in a dialysis membrane against water	*Not explored*	Rojas et al. (2015)
		Two shells of LaPO_4_		80% (precipitation and surface deposition)	> 99.9% at 27 d in a dialysis membrane against water		
α-zirconium phosphate nanoparticles	^223^Ra	–	–	98.5% (surface sorption)	< 1.5% in saline; 14% in bovine plasma at 48 h	*Not explored*	Sakmár et al. (2023)
NaA nanozeolites	^224^Ra	–	–	> 99.9% (ion exchange)	> 99.5% at 24 h in saline, PBS, EDTA, L-cysteine, and human blood serum	*Not explored*	Piotrowska et al. (2013)
Functionalized NaA nanozeolites	^223^Ra	Silane-poly(ethyleneglycol) (PEG)	Substance P (SP) (5–11)	> 99.9% (ion exchange)	> 99.5% at 6 d in human serum at 37 °C (~ 5% release of ^211^Pb and ^211^Bi)	High affinity (*IC*_50_ = 14.3 μg·mL^−1^) to neurokinin 1 (NK-1) receptor on human glioma T98G cells ^[e]^ High cytotoxicity	Piotrowska et al. (2017)
Functionalized NaA nanozeolites	^223^Ra	Silane-PEG	D2B (~ 50 antibodies/ nanoparticle)	99.8%, specific activity 0.65 MBq/mg (ion exchange)	> 95% at 12 d in human serum at 37 °C	Specific binding to PSMA + human lymph node prostate carcinoma LNCaP C4-2 cells ^[f]^ High cytotoxicity Significant accumulation in the spleen, liver, bone, lungs, but not in the tumor, at 7 d in LNCaP C4-2 tumor-bearing BALB/c nude male mice	Czerwińska et al. (2020), Lankoff et al. (2021)
Sterically stabilized PEG-liposomes ^[g]^	^223^Ra	–	Dual: human F(ab’)_2_-folate	78% (ionophore-mediated loading)	93% at 100 h in human serum at 37 °C	High affinity (*K*_a_ ~ 10^9^–10^12^) to folate receptor (FR) on human ovarian carcinoma OvCar-3 (ATTC HTB-161) cells	Henriksen et al. (2004)
Pegylated liposomal doxorubicin (PLD)	^223^Ra	–	–	51–67% (ionophore-mediated loading)	No release at 7 d in fetal calf serum at 37 °C	Significant accumulation in the spleen, skull, and femur at 14 d in healthy white BALB/c mice	Jonasdottir et al. (2006)

^[^^a]^Expressed as percentage of the initially added activity. The method for radionuclide incorporation and/or nanocarrier preparation is reported in brackets

^[b]^Expressed as percentage of the activity retained in the nanocarrier, unless otherwise stated. The specified time point is calculated from the nanocarrier preparation

^[c]^Barium-133 (^133^Ba, *t*_1/2_ = 10.6 y) decays through electron capture to stable caesium-133 (IAEA)

^[d]^Barium-131 (^131^Ba, *t*_1/2_ = 11.5 d - vide infra) (IAEA)

^[e]^Affinity studies were conducted with BaA-silane-PEG-SP(5-11) in competition assays with the iodine-131 (^131^I)-labelled ^131^I-Tyr^8^-SP

^[f]^Binding specificity studies were conducted with ^131^I-D2B-PEG-silane-NaA in competition assays with D2B

^[g]^Composition: 1,2-distearoyl-sn-glycero-3-phosphocholine (DSPC)/cholesterol 2:1 and 5 mol% of N-{ω-[4-(p-maleimidophenyl)butanoyl]amino} poly(ethylene glycol)_2000_ 1,2-distearoyl-sn-glycero-3-phosphoethanolamine, sodium salt (DSPE-PEG_2000_-MPB)

## Imaging with radium: challenges and potential alternatives

^223^Ra can be theoretically considered a theranostic radionuclide thanks to its dual therapeutic (α emission) and diagnostic (γ emission) nature. Quantitative ^223^Ra-SPECT imaging has been reported so far, but the small photon abundance and the scarcity of the activity injected generate low-quality images (Abou et al. 2020; Benabdallah et al. 2019; Herrero Álvarez et al. 2021; Owaki et al. 2017). Nowadays, SPECT acquisitions with ^223^Ra are mainly limited to the evaluation of the activity biodistribution after [^223^Ra]RaCl_2_ therapy (Brito and Etchebehere 2019; Lassmann and Eberlein 2018). As an alternative approach, even if restricted to a preclinical context, Boschi et al*.* (2018) reported the feasibility of detecting ^223^Ra and its short-lived daughters with radioluminescent imaging techniques. The study proved that luminescence imaging can be successfully employed to detect ^223^Ra-biodistribution in murine models, but whether this kind of approach can be applied to a clinic contest is still to be demonstrated.

Several radioisotopes are commonly used in nuclear medicine routine to provide diagnosis of disease, patient selection, and dosimetry measurement. These nuclides include for instance fluorine-18 (^18^F), gallium-68 (^68^Ga), and technetium-99m (^99m^Tc) (Ballinger 2018; Bauckneht et al. 2020; Chakraborty et al. 2013; Dittmann et al. 2021; Filippi et al. 2020; Frantellizzi et al. 2020, 2023; García Vicente et al. 2021; Racaru et al. 2018; Vicente et al. 2017; Wadas et al. 2010). However, due to the significantly different chemistry compared to alkaline earth metals, they cannot act as optimal diagnostic matches for ^223^Ra-based treatments. This issue could be somewhat overcome by employing the available radionuclides of barium that, with a certain grade of approximation, can be considered as a chemical equivalent of radium. Indeed, several barium radioisotopes possess decay properties that make them suitable for imaging purposes. Among these, barium-131 (^131^Ba, *t*_1/2_ = 11.5 d) decays through electron capture (EC) to caesium-131 (^131^Cs, *t*_1/2_ = 9.7 d), which in turn decays to stable xenon-131 via EC (IAEA; Spencer et al. 1970). During its decay, ^131^Ba emits several γ-rays, among which the 123.8 keV (29.8% abundance) and the 216.1 keV (20.4%) radiations can potentially be exploited for SPECT imaging since they are well detected by gamma cameras and provide better image resolution than γ-photons of higher energy (*e.g.* the one at 496.3 keV, 48.0%) (IAEA; Reissig et al. 2020a; Spencer et al. 1970). Another barium radionuclide of clinical interest is barium-135m (^135m^Ba, *t*_1/2_ = 28.7 h). ^135m^Ba decays to stable ^135^Ba via internal transition, by emitting a single γ-ray with 268.2 keV energy and 16.0% abundance (IAEA; Spencer et al. 1970). Since these photons are released nearly without any other accompanying radiation, ^135m^Ba is quite appealing as a possible imaging agent for SPECT (Reissig et al. 2021; Syed and Hosain 1971).

Underlining its similarity with Ra^2+^, Ba^2+^ is also characterized by a calcimimetic behavior and accumulates in the skeleton, particularly in highly metabolically active bone regions (Harrison et al. 1967; Reissig et al. 2020a, 2021). Since long barium is known as a bone-seeking agent and its radioisotopes have been considered for different bone-scanning medical applications, such as quantitative determination of mineral turnover in the bones and scintigraphy of the skeleton (Neirincky 1977; Spencer et al. 1969, 1970). Their potential as radiotracers was foreseen in the late 1960s and has recently been revived for the possibility of providing a diagnostic match for targeted ^223/224^Ra-based radiopharmaceuticals (Harrison et al. 1967; Reissig et al. 2020a).

Finally, the (coordination) chemistry of Ba^2+^ is similar to that of Ra^2+^ and, in most cases, the carrier systems which have been proposed for ^223/224^Ra were studied also with Ba^2+^ (vide supra) (Reissig et al. 2021). As regards the BFC approach, polyamino-polycarboxylic acids like EDTA (Matyskin et al. 2017; Smith and Martell 1987), DTPA (Smith and Martell 1987), and DOTA (Martell et al. 1996) have been considered, supplemented by crown ethers (Lehn and Sauvage 1975), aza-crown ethers (Anderegg 1975; Lehn and Sauvage 1975; Thiele et al. 2018) and calixarenes (Bauer et al. 2018, 2019; Chen et al. 1999; Steinberg et al. 2018). It is worth highlighting that stability and biodistribution of the complex [^131^Ba][Ba(macropa)] displayed similar features with the already described [^223^Ra][Ra(macropa)] thus indicating the applicability of ^131^Ba/^223^Ra as a theranostic pair (Reissig et al. 2020a, 2021).

## Conclusions

[^223^Ra]RaCl_2_ (Xofigo^®^) is the first and nowadays the only FDA- and EMA-approved α-emitter radiotracer. It is largely employed in nuclear medicine to treat skeletal metastases caused by castration-resistant prostate cancer. Nonetheless, a widespread use of therapeutic radiopharmaceuticals labelled with ^223^Ra/^224^Ra is still unlikely unless a method for their stable chelation or incorporation in vivo is found. Several scaffolds, mainly chelators and nanoparticles, have been hitherto explored to bind radium radionuclides and to retain their radioactive daughters with the purpose of selectively delivering the emitted radiation to cancer cells. Unfortunately, no vector combining all these properties has been found to date and much work is still needed to investigate new options prompted to bind Ra^2+^ with sufficient stability for clinical use. A method to selectively carry these radioisotopes to specific tumoral targets would be a remarkable breakthrough able to open the way to the treatment of several cancers with a Ra^2+^-based TAT approach.

## Data Availability

Not applicable.

## Abbreviations

BFC: Bifunctional chelator
Calix-TA: 4-*tert*-Butylcalix[4]arene-tetraacetic acid
CyDTA: Cyclohexane-1,2-diaminetetraacetic acid
DART: Diffusing alpha-emitters radiation therapy
DB30C10: Dibenzo-30-crown-10
DFT: Density functional theory
DOTA: 1,4,7,10-Tetraazacyclododecane-1,4,7,10-tetraacetic acid
DTPA: Diethylenetriaminepentaacetic acid
EDTA: Ethylenediaminetetraacetic acid
EDTMP: Ethylenediaminetetra(methylene) phosphonic acid
EGTA: 2,2′-Ethylenedioxyl bis[ethyl-iminodi(acetic acid)]
EMA: European Medicines Agency
FDA: U.S. Food and Drug Administration
HEDTA: N′-(2-hydroxyethyl)ethylenediamine N,N,N′-triacetic acid
HSAB: Hard and soft acids and bases theory
LET: Linear energy transfer
NTA: Nitrilotriacetic acid
SPECT: Single photon emission computed tomography
PSMA: Prostate specific membrane antigen
TAT: Targeted alpha therapy
